# Deep orofacial phenotyping of population-based infants with isolated cleft lip and isolated cleft palate

**DOI:** 10.1038/s41598-020-78602-w

**Published:** 2020-12-10

**Authors:** Mimi Yow, Nuno V. Hermann, Yuan Wei, Agneta Karsten, Sven Kreiborg

**Affiliations:** 1 Department of Orthodontics, National Dental Centre, SingHealth Duke-NUS Oral Health Academic Programme, 5 Second Hospital Avenue, Singapore, 168938 Singapore; 2 Department of Dental Medicine, Karolinska Institutet, Alfred Nobels Allé 8, Huddinge, Sweden; 3 Department of Pediatric Dentistry and Clinical Genetics, School of Dentistry, Faculty of Health Sciences, University of Copenhagen, Nørre Allé 20, 2200 Copenhagen N, Denmark; 4 Singapore Clinical Research Institute (SCRI), 31 Biopolis Way, Singapore, 138669 Singapore

**Keywords:** Developmental biology, Anatomy, Biomarkers

## Abstract

Isolated orofacial clefts (OFC) are common with poorly understood aetiology. Heterogeneous phenotypes and subphenotypes confound aetiological variant findings. To improve OFC phenome understanding, population-based, consecutive, pre-treatment infants with isolated unilateral cleft lip (UCL, n = 183) and isolated cleft palate (CP, n = 83) of similar ancestry were grouped for deep phenotyping. Subphenotypes stratified by gender and cleft severity were evaluated for primary dental malformations and maturation using radiographs. We found that cleft severity and tooth agenesis were inadequate to distinguish heterogeneity in infants with UCL and CP. Both groups featured slow dental maturity, significantly slower in males and the UCL phenotype. In 32.8% of infants with UCL, supernumerary maxillary lateral incisors were present on the cleft lip side, but not in infants with CP, suggesting a cleft dental epithelium and *forme fruste* cleft dentoalveolus of the UCL subphenotype. The findings underscored the importance of deep phenotyping to disclose occult OFC subphenotypes.

## Introduction

Orofacial cleft (OFC) is the leading birth defect of the craniofacial region and it presents with significant morbidity and health burdens^1^. The prevalence of OFC differs considerably by geographical regions and race, ranging from 2.9 to 23.9 per 10,000^2^. In Denmark, the prevalence is about 20 per 10,000^2,3^. The diverse OFC phenotypes are broadly classified into three principal types: isolated cleft lip (CL) with or without alveolus (cleft of the primary palate only); isolated cleft palate (CP) (cleft of the secondary palate only); and a combination of cleft lip and cleft palate (CLP) (cleft of both the primary palate and secondary palate)^4–6^. The heterogeneous anatomical traits include distinct orofacial and dental characteristics that are of multifactorial origin constituted by genetic, epigenetic and environmental elements. The OFC phenotypes and their spectrum of subphenotypes feature overt and occult traits^7–10^.

The relationship between facial development and odontogenesis is complex. In the developing embryo, the medial nasal and maxillary processes involved in the formation of the primary and secondary palates are interlinked with the development of the dental laminae from which primary teeth develop. In normal foetal development, these processes form the upper lip and the maxillary dental arch^11^. Facial processes fuse by the 38th embryonic day to form the face. Cleft lip is the consequent malformation when these processes are not or are improperly fused^12^. Dental epithelium from the two processes responsible for tooth development in the region of the primary maxillary lateral incisor may be disrupted with non or incomplete fusion^11^. This can affect the maxillary primary lateral incisor that has two origins: a small contribution from the dental lamina in the medial nasal process and a larger contribution from the dental lamina in the maxillary process^13–15^. The defect in the fusion of dental epithelium places the two subcomponents of the primary lateral incisor at risk of forming a spectrum of tooth abnormalities, e.g. supernumeraries, hypoplasia and tooth agenesis, or a subcomponent forms a single normal or microdontic primary lateral incisor^11,16,17^. The reported frequencies of a supernumerary maxillary primary lateral incisor on the cleft side in unilateral clefts of the primary palate vary between 5.5% and 45.2%^16,18–22^. Agenesis of the maxillary primary lateral incisor has been reported to occur in the cleft region in 1.6–14.5% of children with unilateral CL^17,20–22^ and associated delayed dental maturity^23^. The variations in reported frequencies and anomalies can probably be explained by confounders in sampling conditions and bias, methodologies, ethnic groups and treatment.

As the dental epithelium is remote from the tooth-bearing regions in the maxilla, developing teeth should not be involved with defects of the palate in infants with isolated cleft palate (CP). Nevertheless, agenesis of primary teeth adjacent to the cleft dentoalveolus is featured in 1.6% of children with CP^22^. In addition, several studies have reported increased frequencies of agenesis of permanent teeth and delayed maturation of teeth in children with CP^23–29^. Moreover, children with clefts of the secondary palate and agenesis of permanent teeth are found to have the most significant delay in tooth maturation^30,31^.

Dentitional anomalies, in terms of deviations in the number of teeth, malformation of teeth and delayed tooth maturation and eruption, have been reported to affect both the primary and the permanent dentitions, at or remote from the cleft site^17,20,21,32,33^. These characteristics may be integral in the spectrum of defects in non-syndromic OFC. In the primary dentition, supernumerary maxillary lateral incisors in the cleft region are reportedly more common than agenesis of these teeth. In contrast, agenesis of the maxillary lateral incisor is more common than the occurrence of supernumerary teeth in the cleft region of the permanent dentition^20,22^. The reason for this difference remains obscure. Early treatment iatrogenesis may be one of the causes of dentitional anomalies and delayed dental maturation in the maxillary incisal region in infants with CL. Environmental conditions may modify dentitional and maturation traits, especially that in the permanent dentition, which develops postnatally^18,21,24,34,35^.

To remove confounders from sampling bias, treatment iatrogenesis and overlapping malformations in infants with cleft lip and palate, we used population-based samples from Denmark, which had centralised birth registration of Danish infants with cleft malformations for the last 85 years. Stringent selection criteria were used to group the study samples from consecutive live births of non-syndromic pre-treatment Danish infants of Northern European ancestry with isolated cleft lip and isolated cleft palate. Infants with combined lip and palate defects were excluded. This study evaluates dentitional anomalies and deviations of dental maturity by radiographs for deep phenotyping infants with two primary cleft phenotypes, non-syndromic isolated unilateral cleft lip (UCL) and non-syndromic isolated cleft palate (CP).

## Results

### Primary dentition in study and control groups

The control group presented low frequencies of anomalous traits in the primary dentition, below 1% (Table 1).

**Table 1:** UCL group and control: frequencies of dentitional anomalies.

Anomalies	Male UCL	Female UCL	Total UCL	Control
	(N = 118)	(N = 65)	(N = 183)	(N = 4,564)
	N (%)	N (%)	N (%)	N (%)
Agenesis	0 (0.0)	0 (0.0)	0 (0.0)	25 (0.6)
Supernumerary*	42 (35.6)	18 (27.7)	60 (32.8)	26 (0.6)
Microdontia	0 (0.0)	1 (1.5)	1 (0.6)	8 (0.2)
Talon Cusp	3 (2.5)	3 (4.6)	6 (3.3)	0 (0.0)
Fusion	3 (2.5)	0 (0.0)	3 (1.6)	39 (0.9)
Overall	48 (40.6)	22 (33.8)	70 (38.3)	98 (2.1)

12 microdontic supernumerary teeth grouped in Supernumerary*.

There were no anomalies of the primary dentition in all infants with CP, with neither deviation in the number of teeth nor malformations. Tooth agenesis in the primary dentition was not featured in infants with UCL and CP. Even though the control group presented with tooth agenesis, the occurrence of this trait (0.6%) was rare.

**Figure 1:**
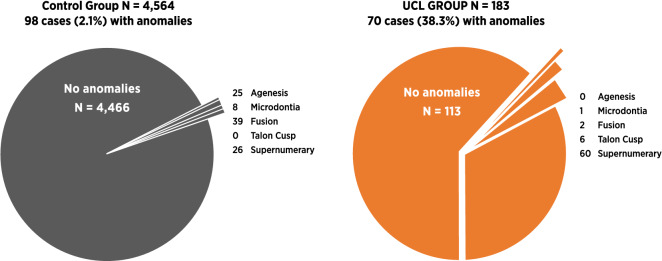
Control and UCL groups: distribution of dentitional anomalies.

**Figure 2:**
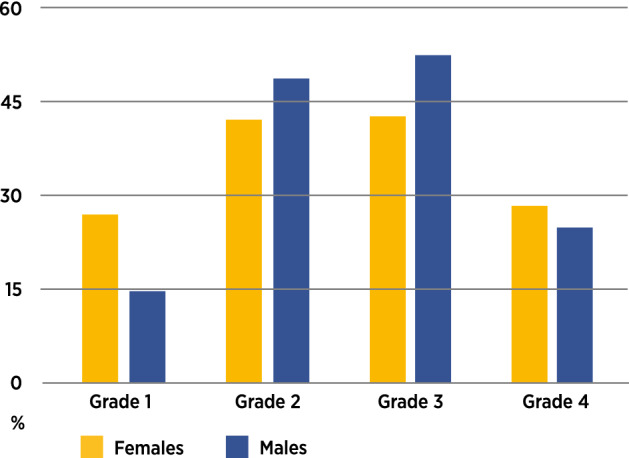
Frequency of dentitional anomalies in infants with UCL: gender and cleft severity.

In infants with UCL, there were findings of anomalies in the primary dentition. These findings and frequencies in infants with UCL were characterised in five distinct groups for comparison with the control (Fig. 1). Both male and female infants with UCL of severity grades 2 and 3 had higher frequencies of dentitional anomalies compared to grades 1 and 4 (Fig. 2).

The risk difference (RD) for overall dentitional anomalies in the UCL phenotype was highly significant when compared with the control group (*p* ≤ 0.0001, RD = 0.36, 95% CI = 0.29,0.43). Within the UCL group, there was no statistically significant risk difference (RD) for overall dentitional anomalies between the genders (*p* = 0.4276, RD = 0.68, 95% CI = 0.077,0.21) (Table 2).

**Table 2:** UCL group and Control: comparative analyses of dentitional anomaly frequencies.

	Male and female comparison			UCL group and control comparison		
Anomalies	*p* value	Risk difference	95% CI	*p* value	Risk difference	95% CI
Agenesis	Nil	0	Nil	0.6238	− 0.0055	− 0.0076, 0.0033
Supernumerary	0.3249	0.079	− 0.060, 0.22	< 0.0001*	0.32	0.25, 0.39
Microdontia	0.3552	− 0.015	− 0.045, 0.015	− 0.2982	0.0037	− 0.0070, 0.015
Talon Cusp	< 0.6676	− 0.021	− 0.079, 0.038	< 0.0001*	0.033	0.0070, 0.059
Fusion	0.5534	0.025	− 0.003, 0.054	0.2191	0.078	0.011, 0.026
Overall	0.4276	0.068	0.077, 0.21	≤ 0.0001*	0.36	0.29, 0.43

*Highly significant risk differences (Fisher’s exact test).

The frequencies of deviations in the number of teeth and teeth with talon cusp were significantly different in infants with UCL compared with the control group: supernumerary tooth formation (*p* < 0.0001, RD = 0.32, 95% CI = 0.25,0.39); talon cusp formation (*p* < 0.001, RD = 0.033, 95% CI = 0.0070,0.059). Nearly all dentitional anomalies in infants with UCL that were observed in the maxilla on the same side as the cleft lip were supernumerary teeth involving 60 maxillary lateral incisors. The exceptions were microdontia, fusion of teeth and talon cusp that featured in 10 infants. Microdontic supernumerary maxillary lateral incisors (n = 12) were located distal to the central maxillary incisor on the side of the cleft lip. Six maxillary lateral incisors with abnormal tooth morphology featured a talon cusp projecting from the palatal surface that also corresponded with the side of the cleft lip. Talon-cusped maxillary lateral incisors were present in 3.3% of the infants with UCL and this characteristic was not observed in a single subject in the control group (n = 4,564). The risk difference for talon cusp formation in infants with UCL compared with the control group was highly significant (*p* < 0.0001, RD = 0.033, 95% CI = 0.0070, 0.059).

The frequency of microdontia that did not involve supernumerary maxillary lateral incisors in infants with UCL was 0.6% compared to a frequency of 0.2% in the control group of non-cleft children. There was low frequency in fusion of teeth. These involved the mandibular canine and lower lateral incisor in infants with UCL (1.6%). The frequency of fusion of teeth in the control group was 0.9%. There were no statistically significant risk differences between infants with UCL and the control group for microdontia and fusion.

The total frequency of dentitional anomalies in infants with UCL was much higher when compared with the control group, 38.3% and 2.1%, respectively. The risk difference (RD) between the infants with UCL and control groups was highly significant (*p* ≤ 0.0001, RD = 0.36, 95% CI = 0.29, 0.43). Looking at the four different cleft severity groups, dentitional anomalies were the most frequent in grades 2 and 3 for both genders. However, the groups with the lowest frequency of dentitional anomalies were polarised in males and females with UCL, grade 1 had the lowest frequency of dentitional anomalies in males, and grade 4 in females (Fig. 2). There was no significant sexual dimorphism in risk difference for dentitional anomalies (*p* = 0.4276, RD = 0.68, 95% CI = 0.077, 0.21) (Table 3).

**Table 3:** UCL & CP Groups: chronological age and dental maturity by gender.

	UCL group						CP group					
	Chronological age in months				Dental maturity in months		Chronological age in months				Dental maturity in months	
	N (%)	Mean (SD)	Median	Range	Median	Range	N (%)	Mean (SD)	Median	Range	Median	Range
Female	65 (35.5)	2.4 (0.4)	2.4	1.3–3.3	1.5	0–7.5	44 (53.0)	2.5 (0.6)	2.4	1.5–4.6	4.5	1.5–4.5
Male	118 (64.5)	2.4 (0.5)	2.3	1.0–4.4	0 (birth)	− 2.5*–7.5	39 (47.0)	2.5 (0.6)	2.3	1.8–4.7	1.5	− 2.5*–4.5
Total	183 (100.0)	2.4 (0.5)	2.4	1.0–4.4	1.5	− 2.5*–7.5	83 (100.0)	2.5 (0.6)	2.3	1.5–4.7	1.5	− 2.5*–4.5

*Equivalent to 30 foetal weeks.

**Figure 3:**
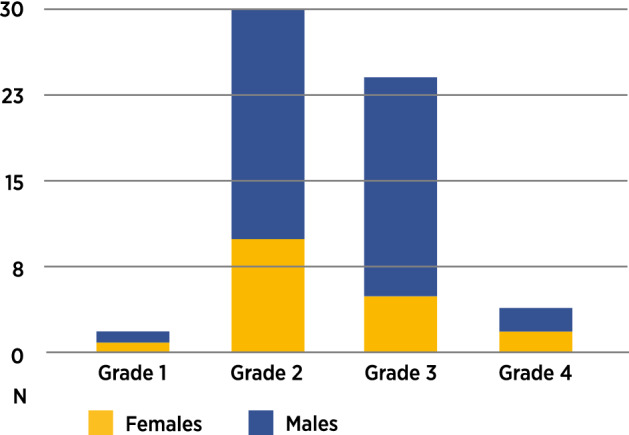
Distribution of supernumerary primary maxillary lateral incisors in infants with UCL: gender and cleft severity.

Sixty of the 183 infants with UCL (32.8%) had supernumerary maxillary primary lateral incisors. All supernumerary teeth were located on the same side as the cleft lip. The frequency of supernumerary primary teeth in the controls was low, 0.6%. In infants with UCL, a supernumerary tooth was more frequent in males (35.6%) than in females (27.7%) although the risk difference in sexual dimorphism for supernumeraries was not statistically significant (*p* = 0.3249, RD = 0.079, 95% CI =  − 0.060,0.22). The paired primary maxillary lateral incisors were located between the maxillary central incisor and canine. The distribution of the 60 supernumerary lateral incisors was most frequent in infants with UCL of grade 2 and 3 severity (Fig. 3).

**Figure 4:**
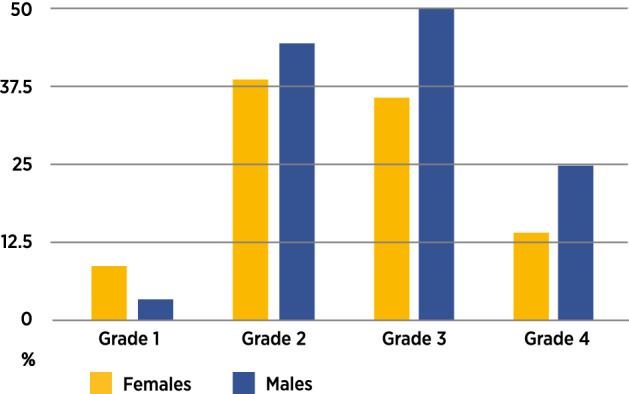
Frequency of supernumerary primary maxillary lateral incisors in infants with UCL: gender and cleft severity.

There was no sexual dimorphism for risk difference in the formation of supernumerary teeth in infants with UCL (*p* = 0.3249, RD = 0.079, 95% CI =  − 0.060,0.22) (Table 2). Half of the supernumerary teeth (n = 30) were observed in the subphenotype with severity grade 2. In contrast, only two infants of subphenotype with severity grade 1 had a supernumerary lateral incisor. The highest frequencies of supernumerary lateral incisors were observed in the subphenotypes with severity grades 2 and 3. For males in subphenotype with severity grade 3, the frequency of a supernumerary lateral incisor was 50%. The lowest frequency was observed in subphenotype with severity grade 1. Most of the supernumerary maxillary lateral incisors (n = 48; 80%) were supplementary lateral incisors and similar in size. The remaining supernumerary maxillary lateral incisors (n = 12; 20%) were microdontic. The frequency of supernumerary maxillary lateral incisors occurring in the UCL phenotype was highest in males with grade 3 cleft severity (Fig. 4).

### Dental maturation

#### Infants with unilateral cleft lip (UCL)

Development stages of 3,720 teeth (including the 60 supernumerary teeth) in UCL were found to fall within six stages of crown and tooth formation in the method of Mooreees^40^. The stages were: coalescence of cusps (Cco); cusp outline complete (Coc); crown half completed with dentine formation (Cr½); crown three-quarters completed (Cr¾); crown completed with defined pulp roof and initial root formation with diverged edges (Ri). Females with UCL had a median chronological age of 2.4 months (range 1.3–3.3 months) and a median dental maturation age of 1.5 months (range 0–7.5 months). Males with UCL had a median chronological age of 2.3 months (range 1.0–4.4 months) and a dental maturation age equivalent to that at birth, 0 months, (range 30 foetal weeks to 7.5 months) (Table 3). The difference in dental maturation age between the genders was highly statistically significant. Males had highly significant delay in dental maturation compared with females (*p* < 0.0001). In the control group, male and female dental maturation ages were within the same age-categories. The delay in dental maturation in infants with UCL was highly significant between the severity grades (*p* < 0.0006) with the greatest delay in grade 2 (Table 4).

**Table 4:** UCL Group: comparison of dental maturity by gender and cleft severity.

Age (month-category)	Female	Male	Grade 1	Grade 2	Grade 3	Grade 4	Total
	N (%)	N (%)	N (%)	N (%)	N (%)	N (%)	N (%)
− 2.5	0 (0.0)	32 (27.1)	11 (28.9)	9 (12.7)	9 (17.3)	3 (13.6)	32 (17.5)
0	1 (1.5)	56 (47.4)	6 (15.8)	27 (38.0)	18 (34.6)	6 (27.3)	57 (31.2)
1.5	44 (67.7)	14 (11.9)	6 (15.8)	21 (29.6)	19 (36.5)	12 (54.5)	58 (31.7)
4.5	19 (29.3)	14 (11.9)	12 (31.6)	14 (19.7)	6 (11.5)	1 (4.5)	33 (18.0)
7.5	1 (1.5)	2 (1.7)	3 (7.9)	0 (0.0)	0 (0.0)	0 (0.0)	3 (1.6)
Total	65 (100.0)	118 (100.0)	38 (100.0)	71 (100.0)	52 (100.0)	22 (100.0)	183 (100.0)

N indicated the number of cases and (%) the frequency of cases.

Highly significant delay in dental maturity in males compared with females (*p* < 0.0001).

Highly significant difference in dental maturity between UCL severity grades (*p* = 0.0006).

Significance testing by Fisher’s exact test.

#### Infants with cleft palate (CP)

Development stages of 1,660 teeth in infants with CP were found to fall within six stages of crown and tooth formation. The stages were: coalescence of cusps (Cco); cusp outline complete (Coc); crown half completed with dentine formation (Cr½); crown three-quarters completed (Cr¾); crown completed with defined pulp roof and initial root formation with diverged edges (Ri). Females with CP had a median chronological age of 2.4 months (range 1.5–4.6 months) and a median dental maturation age of 4.5 months (range 1.5–4.5 months). Males with CP had a median chronological age of 2.3 months (range 1.8–4.7 months) and a dental maturation age of 1.5 months (range 30 foetal weeks to 4.5 months) (Table 3). Highly significant differences in ages for dental maturation were observed between males and females with greater delay in the males (*p* < 0.0001). There were no statistically significant differences in dental maturation ages in infants with CP between the severity grades 1–4 (*p* = 0.2482) (Table 5).

**Table 5:** CP Group: comparison of dental maturity by gender and cleft severity.

Age (month-category)	Female	Male	Grade 1	Grade 2	Grade 3	Grade 4	Total
	N (%)	N (%)	N (%)	N (%)	N (%)	N (%)	N (%)
− 2.5	0 (0.0)	2 (5.1)	0 (0.0)	2 (4.8)	0 (0.0)	0 (0.0)	2 (2.4)
0	0 (0.0)	18 (46.2)	3 (27.3)	12 (28.6)	3 (10.7)	0 (0.0)	18 (21.7)
1.5	5 (11.4)	16 (41.0)	5 (45.4)	8 (19.0)	8 (28.6)	0 (0.0)	21 (25.3)
4.5	39 (88.6)	3 (7.7)	3 (27.3)	20 (47.6)	17 (60.7)	2 (100.0)	42 (50.6)
7.5	0 (0.0)	0 (0.0)	0 (0.0)	0 (0.0)	0 (0.0)	0 (0.0)	0 (0.0)
Total	44 (100.0)	39 (100.0)	11 (13.3)	42 (50.6)	28 (33.7)	2 (2.4)	83 (100.0)

N indicated the number of cases and (%) the frequency of cases.

Highly significant delay in dental maturity in CP males compared with females (*p* < 0.0001).

No significant difference in dental maturity between CP severity grades (*p* = 0.2482).

Significance testing by Fisher’s exact test.

#### Comparison of dental maturation age in UCL and CP

The grades of severity in the groups of infants with UCL and CP did not differ in frequency rankings. In descending order, they were grade 2, grade 3, grade 1, and grade 4, with the most severe grade being the least frequent. There was a significant difference in dental maturation age for different severity grades in infants with UCL (*p* < 0.0006) but no significant difference for infants with different CP severity grades (*p* = 0.2482). Dental maturation age in the group with UCL was significantly delayed compared to the group with CP (*p* < 0.0001) (Table 6).

**Table 6:** UCL and CP groups: comparison of dental maturity.

Age (month-category)	UCL group	CP group
	N (%)	N (%)
− 2.5	32 (17.5)	2 (2.4)
0	57 (31.2)	18 (21.7)
1.5	58 (31.7)	21 (25.3)
4.5	33 (18.0)	42 (50.6)
7.5	3 (1.6)	0 (0.0)
Total	183 (100.0)	83 (100.0)

N indicated the number of cases and (%) the frequency of cases.

Highly significant delay in dental maturity in UCL group compared with CP group (*p* < 0.0001).

Significance testing by Fisher’s exact test.

## Discussion

This is a population-based, radiographic study of dentitional anomalies and dental maturation in consecutive Danish infants of Northern European ancestry with UCL and CP, without any form of surgical intervention. The findings are specific to this population of infants with nonsyndromic UCL and CP, and not generalizable to other populations with different cleft phenotypes. The strengths of this study are in the unbiased sampling from population-based cleft live births, and the stringent selection criteria that set apart confounding factors of gender, race, heterogeneity in cleft severity, and in particular, treatment iatrogenesis.

The ratio of differences in prevalence between infants with UCL and CP was 2.2:1. This concurs with the prevalence of the respective cleft types in global OFC as reported by the International Perinatal Database of Typical Oral Clefts (IPDTOC)^43^. The study samples comprised 94.3% of infants with UCL and 75.5% of infants with CP, which adequately represented the population base of cleft live births. The gender distribution in both groups of infants was also in agreement with the data from IPDTOC^43^. The UCL phenotype was more common in males with a 1.9:1 male to female ratio, which was reversed in the CP phenotype with a male to female ratio of 0.7:1. Laterality or cleft-sidedness in the UCL phenotype featured frequency differences that were 63.4% and 36.6% for left-sided and right-sided clefts, respectively. This was comparable to the reported IPDTOC global average of 63.1% left-sided and 36.9% right-sided clefts^43^. In this study, there was conclusive representation of infants with isolated UCL and isolated CP of the Danish population-based cleft live births.

Subphenotyping by severity of the clefts was carried out by three calibrated orthodontists to establish high reliability in ascertainment^3^. The control data for children without clefts were extracted from the longitudinal findings of 4,564 Danish children comprising 2,327 boys and 2,237 girls between 3 and 3.5 years of age^38^. The children were evaluated over three years by the Copenhagen Municipal Infant Dental Service. Random errors in ascertainment of dentitional anomalies were minimal, if at all, as confirmatory radiographs were done for those detected with dentitional anomalies. The prevalence of anomalies in this control group could, if anything, be anticipated to be higher than that of a population-based sample as the data were from the main municipal treatment centre.

The control data for dental maturity age were from the only available database in the world pertinent to this young age group, the London Atlas for Human Tooth Development and Eruption^39^. In both groups of infants with UCL and CP, dental maturation by tooth development stages was compared with infants without clefts (n = 176) from 28 foetal weeks and up to 9 months postnatal using the norms in the London Atlas^39^. The control group of infants without clefts comprised known age-at-death skeletal and dental remains from the Spitalfields Collection and the Odontological Collection at the Royal College of Surgeons of England in London. The limiting factors in comparability could be differences in dental maturity of dissimilar ancestries in different geographic locations and from different timelines.

In the evaluation of dental maturity in different races and ancestries, the study and control groups were from two countries, Denmark and Great Britain, respectively. This may incur chance findings due to inherent population differences. The Danish and British had mingled ancestries since the eighth century and Denmark presented greater genetic affinity with Great Britain than her neighboring Scandinavian countries and Germany^44,45^. With population admixture, differences in the two populations could have attenuated. Other random errors could be due to different methods of staging tooth maturity in the control and study samples. Digitized roentgencephalograhs may be more sensitive in determining tooth developmental stages than from direct inspection of tooth specimens from foetal or skeletal remains due to technical roentgencephalographic and digital-specific viewing enhancements of the soft tissue dental follicles. This may have resulted in interpreting a much more advanced dental maturity age of the developing teeth than what they appear to be in the roentgencephalographs, indicating an even greater delay in dental maturity of the infants with orofacial clefts. The London Atlas is a tried and tested universal standard for referencing dental maturity of different populations and periods^46,47^.

Ancestral genetics and geographical factors were purported to play a part in odontogenic development and maturity in populations with different races. Those of Caucasian descent were reported to have lower odontogenic potential and slower tooth development^48,49^. In contrast, the London Atlas showed no difference in dental maturity between British children of Caucasian origin and British children of Bangladeshi origin, and no differences in children from eight countries^50–52^. More importantly, there was universality in dental maturity, with little variability, in those aged younger than one year^39^. Timeline differences in samplings of the control and study groups could have resulted in chance findings. Temporal effects, socioeconomic, environmental and nutritional factors had been previously investigated and they did not impact dental development^53,54^.

Agenesis of primary teeth is extremely rare in the general non-cleft population. Likewise, agenesis of primary teeth was not observed in a single infant with either UCL or CP in the present study. It has been argued that, in the developing embryo, with incomplete or lack of fusion of the dental epithelium from the medial nasal process and the maxillary process, the resulting trait would be agenesis of the maxillary primary lateral incisor^11,16,17^. However, studies have reported wide-ranging frequencies from 1.6 to 14.5% in agenesis of this tooth in children with isolated CL^16,17,20,22^. The reason for this discrepancy could be related to sampling conditions and bias or surgical iatrogenesis that could cause reductions of tooth number in treated children with clefts^21^. It seems likely that the true frequency of agenesis in the primary dentition is very low in infants with isolated UCL and isolated CP, and probably comparable to the frequency observed in the general non-cleft population of 0.6%^38^. Sampling bias in this study is minimized by observing a population-based cohort with consecutive, treatment-naïve infants born with clefts in one country of similar ancestry. In the control for dentitional anomalies, there could have been bias towards a higher prevalence of non-cleft anomalies due to cross-sectional sampling of children who sought treatment from the municipal infant dental service. This served to strengthen the significant difference in frequencies of anomalies between children with and without cleft.

The high frequency (32.8%) of a supernumerary maxillary primary lateral incisor in the cleft region of infants with UCL in the present study corroborates with the findings in previous studies^17,22^. There were, however, contrasting low frequencies of 5.5% and 5% reported by Tsai et al.^16^ and Howe et al.^21^, respectively. These differences could have been due to bias in sampling criteria and post-treatment effects. The true frequency of supernumerary primary maxillary lateral incisors in treatment-naïve infants with isolated UCL is close to 33% as shown in this study. The explanation for this high frequency of a supernumerary lateral incisor in the cleft region is possibly from the contributions of dental epithelium from both the medial nasal process and the maxillary process that have the capacity to form a primary lateral incisor separately^11^. The finding of a supernumerary tooth, in the same location, affecting the same tooth, on the same side as the cleft lip malformation, suggests a presumptive dental cleft from incomplete or non-fusion of the dental epithelium in the UCL phenotype and forme fruste cleft alveolus subphenotype.

The occurrence of supernumerary teeth was rare in the mildest form of UCL (grade 1) and highest in the more severe subphenotypes (grade 2 and 3), which was to be expected. The reason for the relatively low frequency of supernumerary teeth in the cleft region in grade 4 was obscure, but this could have been due to the small number of this subphenotype studied.

None of the infants in the CP group had supernumerary teeth, which resembled the findings in the population without cleft^38^. No previous studies on the primary dentition in infants with CP have reported supernumerary teeth. This is probably due to the development of the dental lamina in the dentoalveolar bone being remote from the palate, and hence, unaffected by the palatal malformation.

In infants with UCL with a supernumerary maxillary lateral incisor corresponding to the side of the cleft lip, 20% of the supernumerary lateral incisor were microdontic and positioned distal to the central incisor, whereas the supernumerary maxillary lateral incisor positioned mesial to the primary canine was of normal size in all instances. This finding seems to indicate that the contribution of odontogenic epithelium from the medial nasal process in the embryo to form a primary lateral incisor is less than the contribution from the maxillary process^13^.

Talon cusp presented in six maxillary lateral incisors that corresponded with the side of the cleft lip, and occurred in 3.3% of infants with UCL. This characteristic was not found in the control group. The talon cusp trait occurred most frequently found in the mildest form of UCL (grade 1) and could probably be ascribed to the disturbed fusion of the dental laminae in the cleft region during the embryonic period. As previously suggested by Asllanaj et al.^17^, the grading of cleft severity to subphenotype subjects with CL and CP developed by the method of Jensen et al.^3^ seemed to be relevant and associated odontogenesis with the developmental malformations of OFC. The findings supported deep phenotyping with the primary dentition and its maturation traits as biomarkers of the OFC subphenotypes^17,55^.

For infants in the UCL and the CP groups with a median chronological age of 2.4 and 2.3 months, respectively, the median dental maturation age was 1.5 months. This slow development could be an inherent maturation delay in infants and children with clefts that would normalise later, in adolescence^56^. Feeding challenges and/or infections in the early postnatal period prior to surgical closure of the lip and palate could be other causes in the delayed development^57^. The male UCL and CP phenotypes showed significantly more delayed dental maturation than females with UCL and CP, respectively, that extended to delayed mineralization of teeth^39,58^ in accordance with findings of advanced development in females compared to males. In keeping with sexually dimorphic biological development, the findings in this study showed dental maturation to be highly significant for females to be ahead of males by 1.5–4.5 months in infants with UCL and the CP, respectively.

This study establishes the reference for primary dental maturation ages for Northern European Danish infants born with UCL and CP that is validated by rigorous test–retest for reliability and precision. Population-based and stringent sampling criteria are set to minimise bias and confounding factors of gender, ancestry, cleft heterogeneity, and importantly, treatment iatrogenesis. The subphenotypes in the groups with UCL and CP presents different characteristics in the primary dentition and its dental maturation. Subphenotypic characterizations in tooth patterns and dental maturity are sexually dimorphic and specific to treatment-naïve infants with UCL and CP. Tooth agenesis is not featured in the primary dentition of infants with UCL and CP. Supernumerary teeth are frequently present in infants with UCL but not at all in infants with CP. All supernumerary teeth are associated with the maxillary lateral incisor and are present in about one third of the total group of infants with UCL that coincide with cleft lip laterality. Supernumerary teeth are rare in the least severe cleft type, but frequent in the more severe types of clefts (severity grades 2 and 3).

For both groups of infants with UCL and CP, the dental maturation age is delayed in comparison with the infants’ chronological age. The observed delay in the group with UCL is significantly higher than the group with CP. Dental maturation age is significantly different between the severity grades of infants with UCL but not in infants with CP. There is sexual dimorphism in dental maturation with females in both the UCL and CP groups significantly more advanced than the males.

In this population-based study of treatment-naïve consecutive infants with clefts, we find that cleft severity grades alone are inadequate to distinguish subphenotypic heterogeneity in infants with UCL and CP. Dentitional anomalies, including deviations in the number of primary teeth and malformations of primary teeth are traits that defined the unoperated infants with UCL but not the unoperated infants with CP. Delayed dental maturation in the primary dentition characterises both the unoperated UCL and CP subphenotypes shortly after birth. The findings underscore the importance of deep phenotyping in the accurate capture of phenotypes and subphenotypes that are essential for profiling the OFC phenome in precision medicine^59^.

## Material and methods

All radiographs and records used for this study were from the archives of infants with cleft lip and/or palate in Denmark^3^. The methods were performed in accordance with the relevant guidelines and regulations and approved by the Committees on Health Research Ethics for the Capital Region of Denmark. Protocol number H-16044983 for this research stated no requirement for informed consent was necessary in this study that used retrospective anonymised records and radiographs of consecutive cleft lip and/or palate infants born in Denmark, in the period 1976–1981. The infants’ records were clinical documentation and radiographs obtained soon after birth by three calibrated orthodontists, prior to any surgical intervention^3^. Radiographs were standardised roentgencephalographs in the lateral, frontal and axial projections taken in an infant radiographic machine^36,37^.

### Inclusion criteria

Consecutive Danish infants of Northern European ancestry, born in the period from 1976 to 1981, with non-syndromic unilateral cleft lip, non-syndromic cleft secondary palate and no chromosomal abnormalities with complete records, documentation and radiographs prior to treatment and/or surgery.

### Exclusion criteria

Non-Danish infants, not of Northern European ancestry, born before 1976 and after 1981, with unilateral or bilateral cleft lip and palate, chromosomal abnormalities, syndromes, treatment and/or surgery, and incomplete records, documentation and radiographs.

### Sample demographics

The samples in this study were subsets from 678 consecutive cleft live births in Denmark from 1976 to 1981^3^. Out of a total of 194 cases with isolated unilateral cleft lip (UCL), 183 (94.3%) met the inclusion and exclusion criteria. Of these, 65 were females and 118 were males. The severity of cleft lip was graded using the method of Jensen et al.^3^. The demographics of the sample born with UCL in this study are presented in Table 3. The infants with UCL were subphenotyped according to gender and severity of the cleft lip (cleft lip severity grades 1, 2, 3, and 4) following the method of Jensen et al.^3^, which graded the extent of cleft involvement of the upper lip: grade 1 cleft lip involved up to one-third of the lip height from the lower vermilion border of the upper lip; grade 2 cleft lip involved greater than one-third and up to two-thirds of the upper lip height; grade 3 cleft lip involved greater than two-thirds to subtotal of the upper lip height; grade 4 cleft lip involved the total upper lip height. Out of a total of 110 infants with isolated cleft palate (CP), 83 (75.5%) met the inclusion and exclusion criteria. The group consisted of 44 females and 39 males. The demographics of the infants with CP in this study are presented in Table 3. The 83 infants with CP were subphenotyped according to gender and severity of the cleft palate (cleft palate severity grades 1, 2, 3 and 4) following the method of Jensen et al.^3^, which graded the extent of cleft involvement of the secondary palate: grade 1 cleft palate involved only the soft palate; grade 2 cleft palate involved up to one-third of the palate from the posterior; grade 3 cleft palate involved greater than one-third and up to subtotal of the palate from the posterior; grade 4 cleft palate involved the total length of the palate up to the incisive foramen. A total of 5,320 pre-eruption primary teeth were assessed using 798 roentgencephalograhs (266 in the lateral projection, 266 in the frontal projection and 266 in the axial projection).

### Control data

The Danish control data (Table 1) for non-cleft dentitional anomalies were from a cross-sectional study by the Copenhagen Municipal Infant Dental Service of 4,564 children aged 3–3.5 years. The children were observed over a period of 3 years and those with primary dentition malformations had radiographs to confirm the anomalies^38^. The control data for comparison of dental maturation age involved 145 British foetuses and infants, with known age-at-death and staged for tooth formation and dental maturity age in the London Atlas of Human Tooth Development and Eruption^39^.

### Methods of assessment

Retrospective roentgencephalographs of the head, jaws and teeth with attenuated ionizing radiation to enhance visualisation of the dentofacial structures were used. The average dosage in the infant cephalometer was 0.3 mSv for a set of three roentgencephalographs^37^ in the lateral, frontal and axial projections to constitute radiographic views in three planes. The radiographic films were digitized and viewed with digitally enhanced picture-viewing functions in Windows 10 for better visualisation of the radiographic images of the dentition and dental maturation by tooth stages. Subphenotypes by severity of cleft malformations were determined from cleft registration and clinical records. Dentitional anomalies were described in the clinical records and confirmed by radiographic assessments. The locations and types of dentitional anomalies were recorded.

### Dentitional anomalies

I Deviations in tooth-number: (a) Agenesis—at least one tooth is missing in the normal primary tooth-series of 20 teeth; (b) Supernumerary—at least one tooth is in addition to the normal primary tooth-series of 20 teeth.
II Malformations of the crown: (a) Microdontia; (b) Talon cusp; (c) Tooth-fusion.

### Dental maturation age

Dental maturation age was a categorical value determined by identification of radiographic tooth-stages in the primary tooth-series of each infant’s radiographic tooth-image using the Moorrees developmental tooth stages^40^: stage Ci for initial cusp formation; stage Cco for coalescence of cusps; stage Coc for cusp outline complete; stage Cr½ for crown half completed with dentine formation; stage Cr¾ for crown three-quarters completed; stage Crc for crown completed with defined pulp roof; stage Ri for initial root formation with diverged edges; stage R¼ for root length less than crown length; stage R½ for root length equals crown length. Median dental age stratified by gender was determined from the interactive London Atlas of Human Tooth Development and Eruption at the Uniform Resource Locator (URL): https://www.atlas.dentistry.qmul.ac.uk/index.php?NOLOGIN=TRUE, and checked for confirmation of maturation age between the minimum and maximum tooth formation stages^39^.

The chronological age of infants in the UCL and CP samples was adjusted to reflect premature or late-term births by using a full gestational period of 40 weeks to correct for developmental maturity^41^. Advanced or delayed dental maturation age was the difference in dental age in comparison with chronological age.

### Statistical analysis

Data were collated using Excel for Windows 10 (Microsoft Corp, Redmond, WA, USA). Continuous variables were summarised by descriptive statistics of mean, standard deviation, median, minimum and maximum values, and frequencies and percentages for categorical variables. All statistical analyses were performed with SAS 9.4 (Statistical Analysis System software, SAS Institute, North Carolina, USA). Cohen’s kappa coefficient was used to measure agreement in dental maturity assessments. Fisher’s exact test was used for associations between dental development stages by gender and grades of severity as well as to test statistical differences in frequency of dentitional anomalies between male and female infants with UCL and between the UCL group and controls. All tests performed were two-sided unless otherwise stated. The significance level was set at 5%.

### Error of the study method

The accuracy in establishing dental age for dental maturity was tested for reliability and precision by two orthodontists who independently evaluated the Moorrees developmental tooth stages^40^ on 114 radiographs of 38 infants. One month later, the first orthodontist repeated the evaluation using the same set of radiographs. The intra-assessor and inter-assessors’ determination of dental maturity ages established by Cohen’s kappa coefficient for strengths of agreement were 0.9286 and 0.7994, respectively, which demonstrated almost perfect agreement for reliability and substantial agreement for precision^42^.

### Ethics approval

Protocol number H-16044983 from the Committees on Health Research Ethics for the Capital Region of Denmark.

## Data Availability

Data are available at the following link: https://osf.io/q673a/.

## References

[1] Wehby G, Cassell CH (2010). The impact of orofacial clefts on the quality fo life and healthcare use and costs. Oral Dis..

[2] Grosen D (2010). A cohort study of recurrence patterns among more than 54000 relatives of oral cleft cases in Denmark: support for the multifactorial threshold model of inheritance. J. Med. Genet..

[3] Jensen BL, Kreiborg S, Dahl E, Fogh-Andersen P (1988). Cleft lip and palate in Denmark, 1976–1981: epidemiology, variability, and early somatic development. Cleft Palate J..

[4] Fogh-Andersen P (1942). Inheritance of Harelip and Cleft Palate.

[5] Kernahan DA, Stark RB (1958). A new classification for cleft lip and cleft palate. Plast. Reconstr. Surg. Transplant Bull..

[6] Whitaker LA, Pashayan H, Reichman J (1981). A proposed new classification of craniofacial anomalies. Cleft Palate J..

[7] Marazita M (2007). Subclinical features in non-syndromic cleft lip with or without cleft palate (CL/P): review of the evidence that subepithelial orbicularis oris muscle defects are part of an expanded phenotype for CL/P. Orthod. Craniofac. Res..

[8] Dixon MJ, Marazita ML, Beaty TH, Murray JC (2011). Cleft lip and palate: understanding genetic and environmental influences. Nat. Rev. Genet..

[9] Sharp GC (2017). Distinct DNA methylation profiles in subtypes of orofacial cleft. Clin. Epigenet..

[10] Burg ML, Chai Y, Yao CA, Magee W, Figueiredo JC (2018). Epidemiology, etiology, and treatment of isolated cleft palate. Front. Physiol..

[11] Hovorakova M, Lesot H, Peterka M, Peterkova R (2018). Early development of the human dentition revisited. J. Anat..

[12] Tonge CH (1967). Identification of cell patterns in human tooth differentiation. J. Dent. Res..

[13] Ooé T (1957). On the early development of human dental lamina. Okajimas Folia Anat. Jpn..

[14] Hovorakova M, Lesot H, Peterka M (2005). The developmental relationship between the deciduous dentition and the oral vestibule in human embryos. Anat. Embryol..

[15] Hovorakova M, Lesot H, Peterkova R, Peterka M (2006). Origin of the deciduous upper lateral incisor. J. Dent. Res..

[16] Tsai TP, Huang CS, Huang CC, See LC (1998). Distribution patterns of primary and permanent dentition in children with unilateral complete cleft lip and palate. Cleft Palate-Craniofac. J..

[17] Asllanaj B (2017). Dentition patterns in different unilateral cleft lip subphenotypes. J. Dent. Res..

[18] Bøhn A (1963). The course of the premaxillary nerves and blood vessels. Acta Odontol. Scand..

[19] Hansen K, Mehdinia M (2002). Isolated soft tissue cleft lip: the influence on the nasal cavity and supernumerary laterals. Cleft Palate-Craniofac. J..

[20] Pegelow M, Alqadi N, Karsten AL (2012). The prevalence of various dental characteristics in the primary and mixed dentition in patients born with non-syndromic unilateral cleft lip with or without cleft palate. Eur. J. Orthod..

[21] Howe BJ (2015). Spectrum of dental phenotypes in nonsyndromic orofacial clefting. J. Dent. Res..

[22] Suzuki A (2017). A longitudinal study of the presence of dental anomalies in the primary and permanent dentitions of cleft lip and/or palate patients. Cleft Palate-Craniofac. J..

[23] Pöyry M, Nyström M, Ranta R (1989). Tooth development in children with cleft lip and palate: a longitudinal study from birth to adolescence. Eur. J. Orthod..

[24] Ranta RA (1986). review of tooth formation in children with cleft lip/palate. Am. J. Orthod. Dentofac. Orthop..

[25] Lai MC, King NM, Wong HM (2008). Dental development of Chinese children with cleft lip and palate. Cleft Palate-Craniofac. J..

[26] Hermann NV, Zargham M, Darvann TA, Christensen IJ, Kreiborg S (2012). Early postnatal development of the mandibular permanent first molar in infants with isolated cleft palate. Int. J. Paediatr. Dent..

[27] Hermann NV, Darvann TA, Kreiborg S (2017). Early post-natal development of the mandibular permanent first molar in infants with unilateral complete cleft lip and palate. Orthod. Craniofac. Res..

[28] Hermann NV, Darvann TA, Kreiborg S (2020). Delayed maturation and reduced crown width of the permanent first mandibular molar in all subgroups of cleft lip and palate. Orthod. Craniofac. Res..

[29] Almotairy N, Pegelow M (2018). Dental age comparison in patients born with unilateral cleft lip and palate to a control sample using Demirjian and Willems methods. Eur. J. Orthod..

[30] Ranta RA (1984). Associations of some variables to tooth formation in children with isolated cleft palate. Scand. J. Dent. Res..

[31] Heidelbuchel KL, Kuijpers-Jagtman AM, Ophof R, van Hooft RJ (2002). Dental maturity in children with complete bilateral cleft lip and palate. Cleft Palate-Craniofac. J..

[32] Tannure PN (2012). Prevalence of dental anomalies in nonsyndromic individuals with cleft lip and palate: a systematic review and meta-analysis. Cleft Palate-Craniofac. J..

[33] Rizell S (2020). Scandcleft randomized trials of primary surgery for unilateral cleft lip and palate: dental anomalies in 8-year-olds. Eur. J. Orthod..

[34] Harris EF, Hullings JG (1990). Delayed dental development in children with isolated cleft lip and palate. Arch. Oral Biol..

[35] Fisher DM (2005). Unilateral cleft lip repair: an anatomical subunit approximation technique. Plast. Reconstr. Surg..

[36] Kreiborg S, Dahl E, Prydsoe U (1977). A unit for infant roentgencephalometry. Dentomaxillofac. Radiol..

[37] Hermann NV, Jensen BL, Dahl E, Darvann TA, Kreiborg S (2001). A method for three-projection infant cephalometry. Cleft Palate-Craniofac. J..

[38] Ravn JJ (1971). Aplasia, supernumerary teeth and fused teeth in the primary dentition: an epidemiologic study. Eur. J. Oral Sci..

[39] AlQahtani SJ, Hector MP, Liversidge HM (2010). Brief communication: The London Atlas of human tooth development and eruption. Am. J. Phys. Anthropol..

[40] Moorrees CF, Fanning EA, Hunt EE (1963). Age variation of formation for ten permanent teeth. J. Dent. Res..

[41] Paulsson L, Bondemark L, Söderfeldt B (2004). A systematic review of the consequences of premature birth on palatal morphology, dental occlusion, tooth-crown dimensions, and tooth maturity and eruption. Angle Orthod..

[42] Landis JR, Koch GG (1977). The measurement of observer agreement for categorical data. Biometrics.

[43] IPDTOC (2011). Prevalence at birth of cleft lip with or without cleft palate: data from the International Perinatal Database of Typical Oral Clefts ( IPDTOC ). Cleft Palate-Craniofac. J..

[44] Kershaw J, Røyrvik EC (2016). The ‘People of the British Isles’ project and Viking settlement in England. Antiquity.

[45] Athanasiadis G, Cheng JY, Maillund T (2016). Nationwide genomic study in Denmark reveals remarkable population homogeneity. Genetics.

[46] Alqahtani SJ, Hector MP, Liversidge HM (2014). Accuracy of dental age estimation charts: Schour and Massler, Ubelaker and the London Atlas. Am. J. Phys. Anthropol..

[47] Ghafari R, Ghodousi A, Poordavar E (2019). Comparison of the accuracy of the London atlas and Smith method in dental age estimation in 5–15.99-year-old Iranians using the panoramic view. Int. J. Legal Med..

[48] Polder BJ, van’t Hof MA, van der Linden FPGM, Kuijpers-Jagtman AM (2004). A meta-analysis of the prevalence of dental agenesis in permanent teeth. Community Dent. Oral Epidemiol..

[49] Dhamo B (2018). Ancestry and dental development: a geographic and genetic perspective. Am. J. Phys. Anthropol..

[50] Liversidge HM (2006). Timing of Demirjian’s tooth formation stages. Ann. Hum. Biol..

[51] Liversidge HM (2011). Similarity in dental maturation in two ethnic groups of London children. Ann. Hum. Biol..

[52] Cameriere R, Ferrante L, Liversidge HM, Prieto JL, Brkic H (2008). Accuracy of age estimation in children using radiograph of developing teeth. Forensic Sci. Int..

[53] Conceição ELN, Cardoso HFV (2011). Environmental effects on skeletal versus dental development II: further testing of a basic assumption in human osteological research. Am. J. Phys. Anthropol..

[54] Elamin F, Liversidge HM (2013). Malnutrition has no effect on the timing of human tooth formation. PLoS ONE.

[55] Menezes R, Vieira AR (2008). Dental anomalies as part of the cleft spectrum. Cleft Palate-Craniofac. J..

[56] Tan ELY, Kuek MC, Wong HC, Yow M (2017). Longitudinal dental maturation of children with complete unilateral cleft lip and palate: a case-control cohort study. Orthod. Craniofacial Res..

[57] Jensen BL, Dahl E, Kreiborg S (1983). Longitudinal study of the body height, radius length amd skeletal maturity in Danish boys with cleft lip and palate. Scand. J. Dent. Res..

[58] Stack MV (1960). Forensic estimation of age in infancy by gravimetric observations on the developing dentition. J. Forensic Sci. Soc..

[59] McMurry JA (2016). Navigating the phenotype frontier: the Monarch Initiative. Genetics.

